# Methodological Impact on Curing Kinetics of Bone Cement Based on Poly (Styrene-*co*-Methyl Methacrylate)–2D Nanofiller Nanocomposites

**DOI:** 10.3390/polym17010116

**Published:** 2025-01-05

**Authors:** Mohan Raj Krishnan, Edreese Housni Alsharaeh

**Affiliations:** College of Science and General Studies, AlFaisal University, P.O. Box 50927, Riyadh 11533, Saudi Arabia; mkrishnan@alfaisal.edu

**Keywords:** bone cement, polymer nanocomposite, 2D nanofiller, nano-indentation, elastic modulus, hardness

## Abstract

Herein, we report the methodological impact on the curing kinetics of bone cement based on polymer nanocomposites prepared using different methods. Poly (styrene-*co*-methylmethacrylate)–2D nanofiller nanocomposites (P(S-MMA)–2D Nanofiller) were prepared using bulk and suspension polymerization methods to study the effect of the different methods. The prepared nanocomposites were well-characterized for chemical, thermal, mechanical, and structural characteristics using Fourier Transform Infrared spectroscopy (FT-IR), differential scanning calorimetry (DSC), nano-indentation, and scanning electron microscopy (SEM) techniques, respectively. The FT-IR results confirmed the successful formation of the polymer nanocomposites. The DSC results showed that the prepared nanocomposites have higher thermal stabilities than their copolymer counterparts. The nano-indentation results revealed that the elastic modulus of the copolymer nanocomposites (bulk polymerization) was as high as 7.89 GPa, and the hardness was 0.219 GPa. Incorporating the 2D nanofiller in the copolymer matrix synergistically enhances the thermo-mechanical properties of the bone cement samples. The polymer nanocomposites prepared using the suspension polymerization method exhibit faster-curing kinetics (15 min) than those prepared using the bulk polymerization (120–240 min) method.

## 1. Introduction

In the late 1930s, it was discovered that when a powder of polymethylmethacrylate (PMMA) is mixed with methylmethacrylate (MMA) monomer along with an initiator (benzoyl peroxide, BPO), it produces a dough that can be shaped into any desired form using plaster molds and subsequently polymerized into a solid mass [1,2,3,4,5,6]. This polymer powder (solid) and monomer (liquid) two-component system was initially used as a filler for cranial defects [7,8]. The successful commercial production of PMMA during the same period extends its utilization as a denture base and prosthetic material [9,10]. PMMA is a highly transparent and mechanically stable polymer. Therefore, PMMA has been used as a potential bone cement material since the 1930s. Shortly after, it was also found that PMMA with enhanced molding characteristics can be obtained using a mixture of PMMA powder and their microbeads [11]. PMMA-based bone cement is more commonly known as acrylic bone cement. Since its discovery, acrylic bone cements have been successfully used to fix total joint replacement prostheses to periprosthetic bone [12,13,14,15,16]. The importance of bone grafts in successful implant treatments can be found elsewhere [17,18]. Generally, bone cement consists of a solid component, which is usually a mixture of PMMA powder, an initiator, and a radiopacifier; and a liquid component, which is a mixture of MMA monomer, room-temperature or “cold” activator (dimethyl-p-toluidine, DMPT), and an inhibitor (hydroquinone, HQ) [4,19].

The chemical composition, viscosity, amount of radiopacifier, antimicrobial agents, other chemical additives, mixing methods, and temperature during mixing are key factors affecting the mechanical properties, biocompatibility, and clinical performance of acrylic bone cement [20,21,22,23,24,25,26,27,28,29,30]. Note that there is always a mechanical mismatch between the human bone and the PMMA-based bone cement implants. This crucial issue can be successfully addressed by the incorporation of nanostructured materials such as carbon nanotubes (CNT), graphene (G), and graphene oxide (GO) in bone cement formulations, which would enable the fine-tuning of mechanical properties of the bone implants closer to that of human bones [24,30,31,32,33,34]. Also, adding silver (Ag) nanoparticles into bone cement brings anti-microbial functionality [35,36,37]. The interaction of nanosilver with cells and its issues related to cytotoxicity can be found in our previous studies [38,39]. Similarly, adding nanohydroxyapatite (nHA) to bone cement promotes biocompatibility [40,41,42,43,44,45]. Therefore, PMMA-based polymer nanocomposites with improved functionalities could replace PMMA alone bone cement in the future bone cement market. Moreover, the slow polymerization kinetics of PMMA can cause the toxic MMA monomer to diffuse easily into other body parts, leading to further complications for the patient [46,47,48,49]. For instance, potential leaching of the residual monomer into the bloodstream has been reported to be a major contributing factor to the development of cardio-respiratory and cardiovascular complications [50,51]. In addition, the polymerization of PMMA is exothermic and can cause heat-related complications at the surgical site [52,53,54]. In many cases, high temperatures have caused bone necrosis and tissue damage, which eventually result in failed prosthetic fixings [55]. Also, during polymerization, residual stress can develop in situ, leading to microcracks within the cement and gaps at the implant’s interface, which can lead to premature implant failure [11,56]. Therefore, it is crucial to decrease the heat release during the polymerization of MMA and increase the polymerization rate; identifying the monomer conversion, polymerization stress, and polymerization exotherm is important for evaluating the monomers’ performance and controlling the curing process.

Recently, Krishnan et al. reported a detailed study on the polymerization kinetics of multi-functional bone cement based on poly (styrene-methyl methacrylate)–2D nanofiller nanocomposites [39]. The extent of heat generated during polymerization can be successfully overcome by carrying out the polymerization of MMA in the presence of pre-polymerized PMMA–2D nanofiller nanocomposites. However, the slow polymerization rate of PMMA is still a challenge. Therefore, this study aims to systematically determine the effect of the polymerization method of polymer nanocomposites and their respective effect on the polymerization kinetics of bone cement. For that, the polymer nanocomposites were prepared using bulk and suspension polymerization methods. Poly (styrene-methyl methacrylate)–2D nanofiller nanocomposites (P(S-MMA)–2D nanofiller nanocomposites) with different nanofillers, such as commercial graphene (CG), boron nitride (BN), and a 1:1 wt.% mixture of CG and BN (CG:BN) in two different polymerization techniques, viz. bulk, and suspension methods to determine the effect of the polymerization method on the polymerization (setting or curing) kinetics of the bone cement samples.

## 2. Experimental Methods

### 2.1. Materials

Styrene (S, M_w_ = 104.15 g/mol, >99% purity) and methylmethacrylate (MMA, M_w_ = 100.12 g/mol, 99% purity) were procured from Sigma Aldrich. Free-radical initiators such as N, N’ azoisobutyronitile (AIBN, M_w_ = 164.21 g/mol), and benzoyl peroxide (BPO, synthetic grade with an assay of 72.0–80.0%) were obtained from Aldrich. Commercial graphene (CG) was purchased from XG Sciences (XGnP Graphene Nanoplatelets), and h-BN was purchased from SRL Chem (surface area = 19.4 m^2^/g, with an average platelet size of 70 nm). Methanol and ethanol were purchased from Macron Fine Chemicals with a purity of 99.8%. The monomers and solvents were used as received. The initiators were recrystallized using methanol. The CG: BN composite nanofillers were prepared by mixing 1:1 wt.% of CG and BN, followed by ball-milling (SPEX Sample Prep Mixer/Mill 8000M, Cole-Parmer, 65 Liberty Street, Metuchen, NJ 08840, USA) for 45 min.

### 2.2. Preparation of Poly (Styrene-co-Methylmethacrylate)–2D Nanofiller Nanocomposites

#### 2.2.1. Bulk Polymerization

To prepare the P(S-MMA)–2D nanofiller nanocomposite by bulk polymerization technique, 0.01 wt.% of 2D nanofiller (CG or BN or CG: BN) was thoroughly mixed with a 1:1 wt.% of a monomer mixture of styrene and methylmethacrylate by sonication for 30 min at ambient conditions. After the complete mixing of the 2D nanofiller in the monomer mixture, 0.01 wt.% of AIBN was added, and the temperature of the mixture was raised to 70 °C and kept for 48 h. for the completion of polymerization. After that, the formed P(S-MMA)–2D nanofiller composite was washed with hot methanol (60 °C) followed by hot ethanol (60 °C) several times to remove any unreacted monomers, oligomers, and homopolymers. Then, the nanocomposite was dried in a vacuum oven. The nanocomposite powder was prepared by ball-milling the samples for one hour. P(S-MMA) samples were prepared similarly without adding the 2D nanofiller. A detailed experiment for preparing the polymer nanocomposites can be found in our previous reports [57,58,59,60,61,62,63].

#### 2.2.2. Suspension Polymerization

To prepare the P(S-MMA)–2D nanofiller nanocomposite by the suspension polymerization technique, 0.1 g of sodium dodecyl sulfate (SDS) was dissolved in 100 mL of deionized (D.I.) water in a 3-necked round-bottom (RB) flask kept in an oil bath with a thermostat and magnetic stirrer. A condenser with a continuous water flow was connected to the RB flask. After the complete dissolution of the SDS in water, a mixture of 1:1 wt.% of S and MMA, 0.01 wt.% of 2D nanofiller (CG or BN or CG: BN), and 0.01 wt.% of AIBN or BPO was added to the RB flask drop-wise while the reaction mixture was stirred at 1400 rpm. Then, the temperature of the reaction mixture was increased to 70 °C and kept for two hours. Afterward, the temperature of the reaction mixture was increased to 80 °C for six hours and 90 °C for another six hours. Then, the formed nanocomposite samples were thoroughly washed with hot methanol (60 °C) and hot ethanol 60 °C several times to remove the SDS and other byproducts of the reaction. The samples were dried in a vacuum oven.

#### 2.2.3. Preparation of the Bone Cement

A typical bone cement package consists of a solid powder and liquid components. The solid powder is usually a mixture of polymer, initiator, and other additives. The liquid component is typically a mixture of monomer(s), room-temperature activator, and auto-initiation inhibitor. In our study, the solid powder consisted of 98.4 wt.% of P(S-MMA)–2D nanofiller nanocomposite powder and 1.6% benzoyl peroxide (BPO). The liquid component consists of 99.9 wt.% of the MMA monomer and 0.1 wt.% di-methyl-p-toludine (DMPT, activator). To determine the curing kinetics, the solid powder and liquid components were mixed until the sample was completely hardened. The curing kinetics of bone cement are analyzed as dough time and curing time. Dough time is the duration of the process from the beginning of mixing the solid and liquid components to the hardening of the cement; at that point, no more mixing or shape changes are possible (the mixture is no longer a dough). Curing time is the duration between the beginning of mixing the solid and liquid components into a completely hardened cement (final bone cement) [39].

#### 2.2.4. Method of Mixing

The method of mixing is crucial in preparing bone cement with reproducible properties. Our bone cement preparation method involves an instantaneous addition of the liquid component to the solid powder and subsequent fast mixing using a glass stirrer without forming any air bubbles.

### 2.3. Characterizations

#### 2.3.1. Scanning Electron Microscopy (SEM)

The cross-sectional morphologies of the samples were analyzed using FE-SEM (FEI Inspect S50). The samples were coated with Au nanoparticles (2 nm thick) using the sputter-coating method to avoid charging of the samples. The SEM images were recorded at an accelerating voltage of 2 kV.

#### 2.3.2. Fourier Transform Infrared Spectroscopy (FT-IR)

FT-IR spectra of the samples were measured using the Thermo Scientific Nicolet-iS10 instrument, 168 Third Avenue. Waltham, MA, USA. The measurements were made in the wavenumber range of 4000–500 cm^−1^. The FT-IR spectra of the samples were collected using ATR mode.

#### 2.3.3. Differential Scanning Calorimetry

The differential scanning calorimetric (DSC) measurements of the samples were carried out using Hitachi STA7200, Toranomon, Minato-ku, Tokyo 105-6409, Japan. The samples were analyzed in the temperature range of −20 °C to 250 °C at a heating rate of 5 °C/min under a nitrogen flow of 50 mL/min. Approximately 5 mg of sample was used for each measurement.

#### 2.3.4. Nanoindentation Tests

A NanoTestTM system (Micro Materials, UK, Unit D, Willow House, Willow House, Yale Business Village, Ellice Way, Wrexham LL13 7YL, UK) indenter was used to measure the elastic modulus (E) and hardness (H) of the samples using a standard diamond Berkovich. Loading and unloading cycles were kept for 10 s, and the dwell time at each peak load was 5 s. The E and H values were calculated from the force-displacement (*P-h*) profiles. The measurements were conducted at 25 °C in a temperature-controlled environment. At least five measurements were carried out on each sample in 0.1 mN loads.

## 3. Results and Discussion

### 3.1. Preparation of P(S-MMA)–2D Nanofiller Nanocomposites by Bulk and Suspension Polymerization

**Figure 1:**
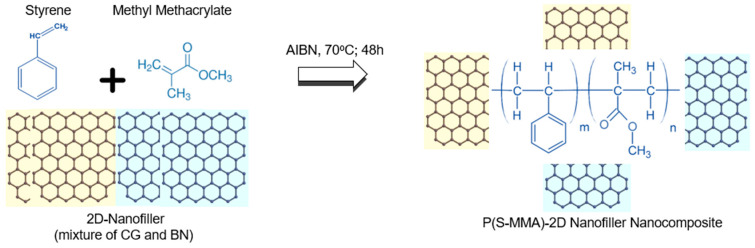
Preparation of P(S-MMA)–2D nanofiller nanocomposites.

In this study, the P(S-MMA)–2D nanofiller nanocomposite samples were prepared using bulk and suspension polymerization techniques. In the bulk polymerization method, both the monomers (S and MMA) in equal ratios (1:1 by wt.) were mixed with a specific amount of 2D nanofiller (CG or BN or CG:BN (0.01 wt.%)) along with the initiator, AIBN (0.01 wt.%). When the reaction mixture was heated to a specific temperature (above the decomposition temperature of the AIBN, 70 °C), the initiator molecules underwent decomposition and produced free radicals (I^•^). Using the initiator free radicals, the monomer molecules were also converted into free radicals. Once the monomer free radicals (I-S^•^ and I-MMA^•^) are generated, the monomer molecules randomly react with the free radicals ((S-MMA)_m_^•^), and chain propagation takes place. After the chain propagation completes, the polymerization reaction concludes by disproportionation ((S-MMA)_m_) or by coupling reaction ((S-MMA)_2m_) of the polymeric chain radicals. Since the 2D nanofillers have chemically reactive linkages (−C=C− or −B=N−), they also actively participate in the ongoing polymerization, resulting in their respective random P(S-MMA)–2D nanofiller copolymer nanocomposites (Figure 1).

In the suspension polymerization technique, the water-insoluble monomers (S and MMA) along with the initiator 2D nanofillers are heterogeneously dispersed in water by continuous stirring, and the monomeric mixture droplets are stabilized by a surfactant such as SDS. When the reaction mixture is heated to a temperature above 70 °C, the initiator molecules are decomposed into free radicals, and in turn, monomeric free radicals start to form. Over time, the monomeric 2D nanofiller droplets are converted into polymeric nanocomposite powder in the water medium by a free radical polymerization mechanism.

### 3.2. Preparation of Bone Cement Using P(S-MMA)–2D Nanofiller Nanocomposites

#### 3.2.1. Room-Temperature Activation of BPO by DMPT

**Figure 2:**
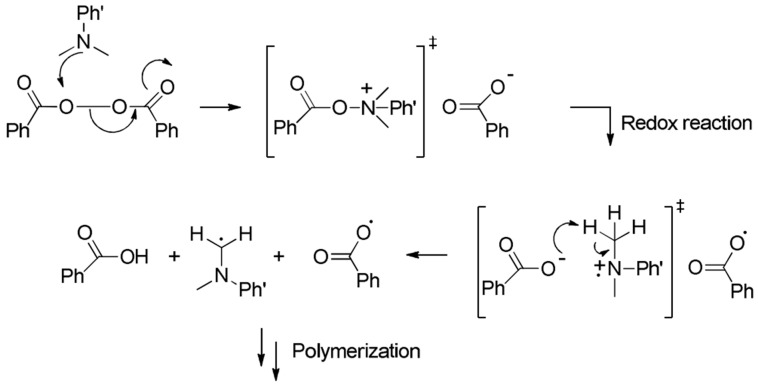
Room-temperature activation of BPO initiator with DMPT [64].

To prepare the bone cement, a specific amount of solid components consisting of P(S-MMA)–2D nanofiller composite powder and initiator (BPO) to be mixed with a liquid component, which is made up of MMA monomer and room temperature activator, DMPT. The room-temperature activation mechanism of BPO by DMPT is a complex reaction sequence with a nucleophilic attack of the tertiary amine on the peroxide bond of the BPO molecule followed by a redox reaction and formation of a benzoyloxy and an anilinomethyl radical that is a carbon-centered radical derived from the tertiary amine, as shown in Figure 2 [64].

#### 3.2.2. Curing Kinetics of Bone Cement

**Figure 3:**
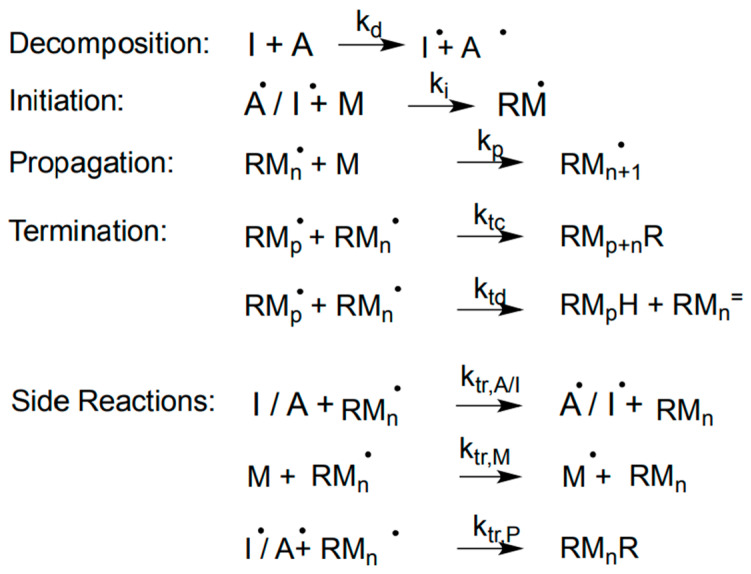
Curing kinetics of bone cement. I is the initiator; A is the activator; M is the monomer; RM^•^ is formed monomer radical by reacting with the initiator radical; k_d_, k_i_, k_p_, and k_t_ are the respective rate constants of initiator decomposition, initiation, propagation, and termination reactions [64].

The room-temperature curing mechanism of the bone cement is clearly depicted in Figure 3 [64]. When the solid and liquid components are mixed, the initiator, BPO (I), undergoes decomposition with the activator DMPT (A), resulting in I^•^ and A^•^ radicals, as shown in Figure 3. Then, the I^•^ and A^•^ radicals react with monomer (M) to form RM^•^ (R can be either I or A radical units). Subsequently, the RM^•^ undergoes chain propagation reactions and forms RM_n_^•^, RM_p_^•^, and so on, followed by a termination reaction either by a combination (RM_n + P_R) or disproportionation (RM_n_H). In addition to this main reaction, there are some side reactions that also take place simultaneously. The RM_n_^•^ reacts with I and A to form RM_n_ and I^•^ and A^•^ radicals. Also, the RM_n_^•^ reacts with M to form RM_n_ and M^•^. At the same time, RM_n_^•^ can also react with I^•^/A^•^ to form RM_n_R.

### 3.3. FE-SEM

**Figure 4:**
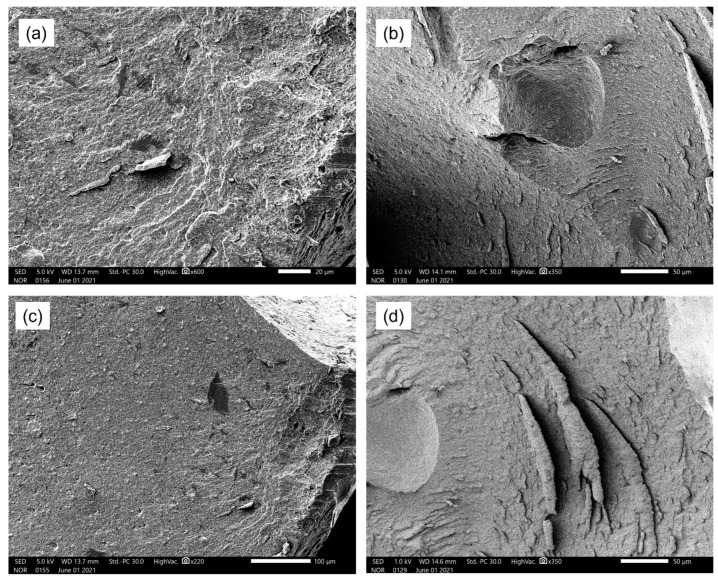
SEM images of bone cement prepared by suspension polymerization. (**a**) P(S-MMA), (**b**) P(S-MMA)/CG, (**c**) P(S-MMA)/BN, and (**d**) P(S-MMA)/CG:BN.

**Figure 5:**
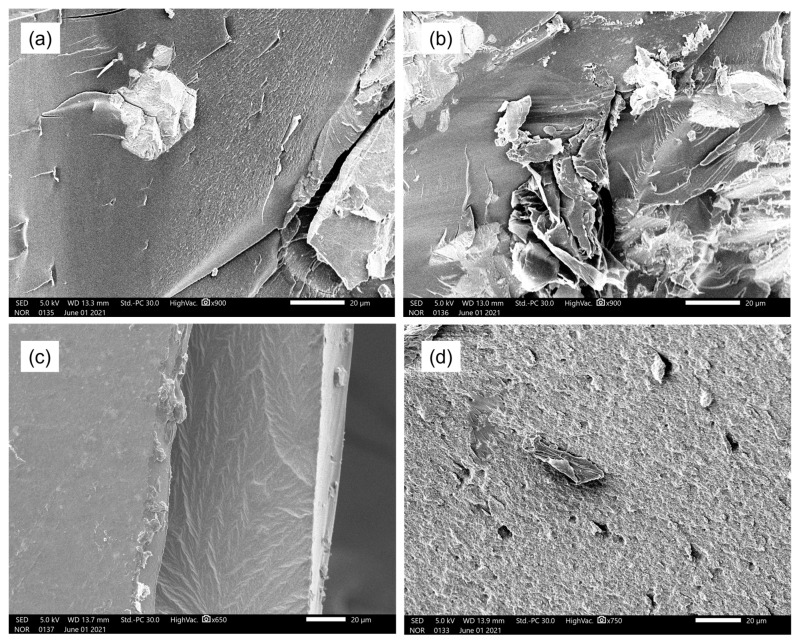
SEM images of bone cement prepared by bulk polymerization. (**a**) P(S-MMA), (**b**) P(S-MMA)/CG, (**c**) P(S-MMA)/BN, and (**d**) P(S-MMA)/CG:BN.

Figure 4 and Figure 5 show the cross-sectional images of bone cement samples (P(S-MMA); P(S-MMA)/CG; P(S-MMA)/BN; P(S-MMA)/CG:BN) that are prepared using the polymer nanocomposites synthesized by suspension and bulk polymerization, respectively. As evident from the FE-SEM images, the bone cement samples exhibit non-porous structures that are irrelevant to their polymerization method. Note that the mechanical properties of the bone cements are inversely related to their porous structures, i.e., the higher the porous structure, the lower the strength of the bone cement samples. However, the bone cement samples that were prepared using the polymer nanocomposites and synthesized using either bulk or suspension polymerization show no porous structures. Graphene is a flat monolayer of carbon atoms organized in hexagonal cells. Similarly, BN is a flat monolayer of B and N atoms organized in hexagonal cells. All the 2D nanofillers (CG; BN and CG:BN) used in the study have identical morphologies. Therefore, the bone cements prepared using different nanofillers show similar morphology. Hence, they should have higher mechanical properties, which is one of the crucial properties of bone cement for their successful applications. Moreover, no distinct difference has been noted with the bone cement samples with different 2D nanofillers such as CG, BN, and CG:BN.

### 3.4. FT-IR

**Figure 6:**
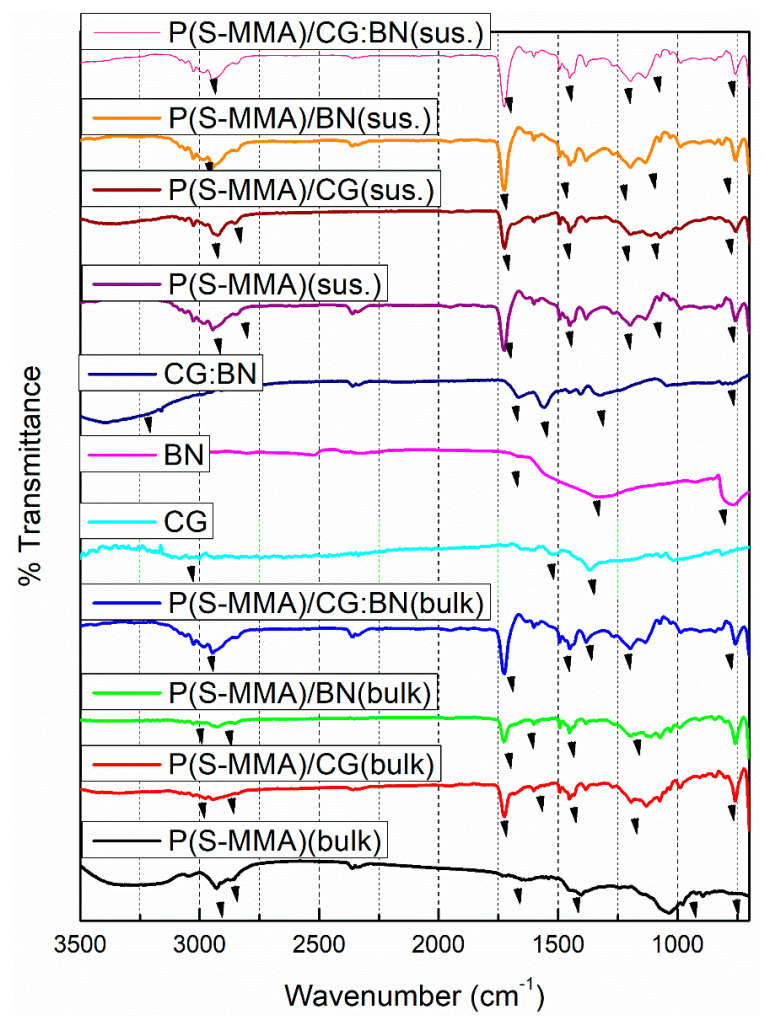
FT−IR spectra of P(S-MMA)–2D nanofiller nanocomposite bone cement samples prepared using bulk and suspension polymerization methods.

Figure 6 shows the FT-IR spectra of P(S-PMMA); P(S-PMMA)/CG; P(S-PMMA)/BN; P(S-PMMA)/CG:BN nanocomposites that were prepared using both bulk and suspension polymerization in addition to the 2D nanofillers of CG; BN; and CG:BN. The P(S-MMA) samples prepared by bulk or suspension polymerization exhibited peaks at 691 cm^−1^, which is related to the mono-substituted ring in-phase bending in PS, at 784 cm^−1^, which is related to the asymmetric band of C-C-O group in PMMA, at 1030 cm^−1^, which is related to the stretching mode of C-O-C, at 1400 cm^−1^ and 2929 cm^−1^, which are related to bending and stretching modes of the –CH_3_ group, respectively, at 1646 cm^−1^, which is related to the stretching mode of C=C bond in the PS), and at 1758 cm^−1^, which is related to the C=O in the PMMA. The characteristic peaks of graphene were identified at 1084 cm^−1^, 1387 cm^−1^, 1627 cm^−1^, and 1726 cm^−1^. Similarly, characteristic peaks of BN were detected at 800 cm^−1^, 1160 cm^−1^, and 1400 cm^−1^. The combination of the peaks was identified for the CG:BN nanocomposites. For the copolymer nanocomposites, the peaks corresponding to both the copolymer (P(S-MMA)) and the respective 2D nanofillers (CG; BN; and CG:BN) were also detected in the respective nanocomposites with slight peak shifts from their original positions [59,65]. The interaction of the 2D nanofillers (CG and BN) with polymeric chains of in P(S-PMMA) was discussed in detail in our previous reports [59,63,66]. The FT-IR results confirmed the successful copolymer nanocomposite formation. Moreover, the FT-IR results also indicated that the copolymer nanocomposite samples that were prepared using both bulk and suspension polymerization are chemically the same.

### 3.5. Thermal Properties

**Figure 7:**
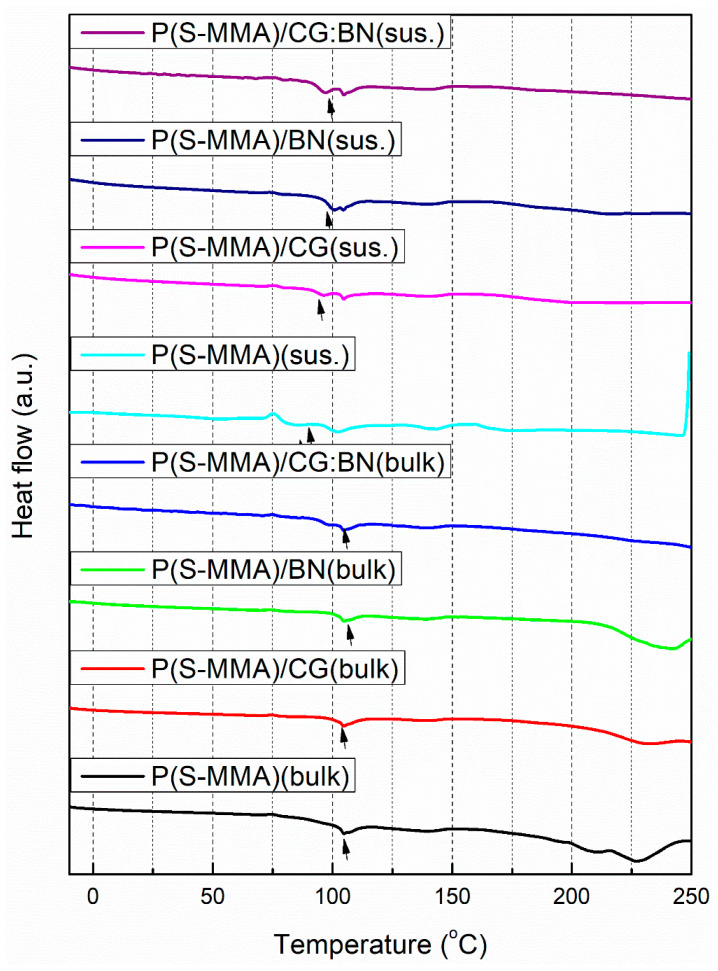
DSC curves of P(S-MMA)–2D nanofiller nanocomposite bone cement samples prepared using bulk and suspension polymerization methods.

Figure 7 shows the DSC curves of P(S-MMA)–2D nanofiller nanocomposite bone cement samples prepared using both bulk and suspension polymerization methods. The glass transition temperatures (T_g_) of the samples are given in Figure 7. As evident from the DSC curves, the T_g_ of P(S-MMA), P(S-MMA)/CG, P(S-MMA)/BN, and P(S-MMA)/CG:BN prepared using bulk polymerization methods are 105.4 °C, 105.1 °C, 105.9 °C, and 106.4 °C, respectively (Table 1). Therefore, the observed T_g_ values of the samples are arranged in decreasing order as P(S-MMA) CG:BN > P(S-MMA)/BN > P(S-MMA) > P(S-MMA)/CG in the bulk polymerization method. The lower T_g_ of P(S-MMA)/CG in comparison to that of P(S-MMA) is due to the lubricating effect of CG nanosheets (acting as a flexiblizer) in the polymer matrix [65,66,67,68,69,70]. However, the copolymer composite with BN has a higher T_g_ value than that of the copolymer. This is due to the interaction of anchoring groups of the BN nanosheets within the polymer matrix. Interestingly, the copolymer nanocomposite samples with a mixture of CG:BN exhibit the highest T_g_ value among all other nanocomposite samples and their copolymer counterparts [61,63]. This thermal enhancement is due to the synergistic effect of the CG and BN nanofillers, which we have explained in previous reports.

Similarly, the T_g_ of the P(S-MMA), P(S-MMA)/CG, P(S-MMA)/BN, and P(S-MMA) CG:BN samples prepared using suspension polymerization methods are 89.2 °C, 98.6 °C, 99.3 °C, and 100.0 °C, respectively (Table 1). These results indicated that the copolymer nanocomposites prepared using bulk polymerization methods show higher T_g_ values than those prepared using suspension polymerization. This can be credited to the relatively longer chain lengths and higher molecular weight of the polymers resulting from bulk polymerization. In suspension polymerization, the polymers’ chain lengths and molecular weight might be lower as the polymerization occurred in dispersed microdroplets of the monomers in a water medium.

### 3.6. Nanomechanical Properties

**Figure 8:**
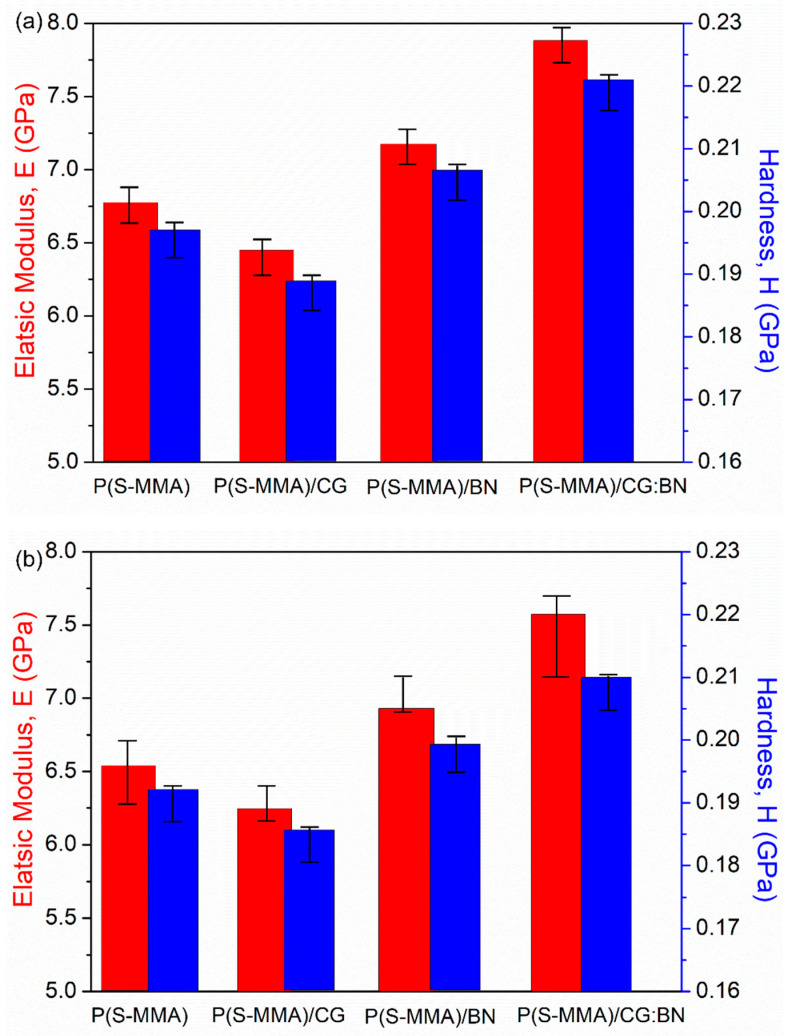
Nanomechanical of P(S-MMA) and P(S-MMA)–2D nanofiller nanocomposites that are prepared using (**a**) bulk and (**b**) suspension polymerization methods.

The elastic modulus (E) and hardness (H) values of the bone cement samples were determined by the nanoindentation technique, and the values are summarized in Table 2. The elastic modulus of P(S-MMA), P(S-MMA)/CG, P(S-MMA)/BN, and P(S-MMA)/CG:BN prepared using bulk polymerization methods are 6.62 GPa, 6.46 GPa, 7.23 GPa, and 7.89 GPa, respectively (Figure 8). The hardness values of P(S-MMA), P(S-MMA)/CG, P(S-MMA)/BN, and P(S-MMA)/CG:BN prepared using the bulk polymerization methods are 0.196 GPa, 0.189 GPa, 0.205 GPa, and 0.219 GPa, respectively. Therefore, E and H values of the samples are arranged in decreasing order as P(S-MMA) CG:BN > P(S-MMA)/BN > P(S-MMA) > P(S-MMA)/CG in the bulk polymerization method. The lower E and H of P(S-MMA)/CG than that of P(S-MMA) result from the lubricating effect of CG nanosheets within the copolymer matrix [65,66,67,68,69,70]. Nevertheless, the P(S-MMA)/BN has higher E and H values than that of the P(S-MMA). This is credited to enhanced physical interaction via the anchoring groups of the BN nanosheets in the polymer matrix. Also, the P(S-MMA)/CG:BN exhibits the highest E and H values among all other nanocomposite bone cements. This mechanical enhancement is credited to the synergistic effect of the CG and BN nanofillers.

At the same time, the E values of the P(S-MMA), P(S-MMA)/CG, P(S-MMA)/BN, and P(S-MMA) CG:BN samples prepared using suspension polymerization methods are 6.52 GPa, 6.36 GPa, 7.12 GPa, and 7.54 GPa, respectively (Figure 8), and the H values of the P(S-MMA), P(S-MMA)/CG, P(S-MMA)/BN, and P(S-MMA) CG:BN are 0.191 GPa, 0.181 GPa, 0.197 GPa, and 0.209 GPa, respectively. These results indicated that the copolymer nanocomposites prepared using bulk polymerization methods show higher E and H values than those prepared using suspension polymerization. Bulk polymerization generally results in relatively longer polymer chains, and the polymers have higher molecular weights [61,63]. On the contrary, suspension polymerization results in a shorter polymer chain and low molecular weight as the polymerization takes place in the microdroplets of the monomers. The chain length and the higher molecular weights of the polymers are the key factors for the higher mechanical strengths of the samples. Therefore, the bone cement samples that are prepared using bulk polymerization exhibit higher nanomechanical properties. Moreover, the nanomechanical properties of the samples are in line with their respective thermal properties.

### 3.7. Curing Kinetics of Bone Cement

**Figure 9:**
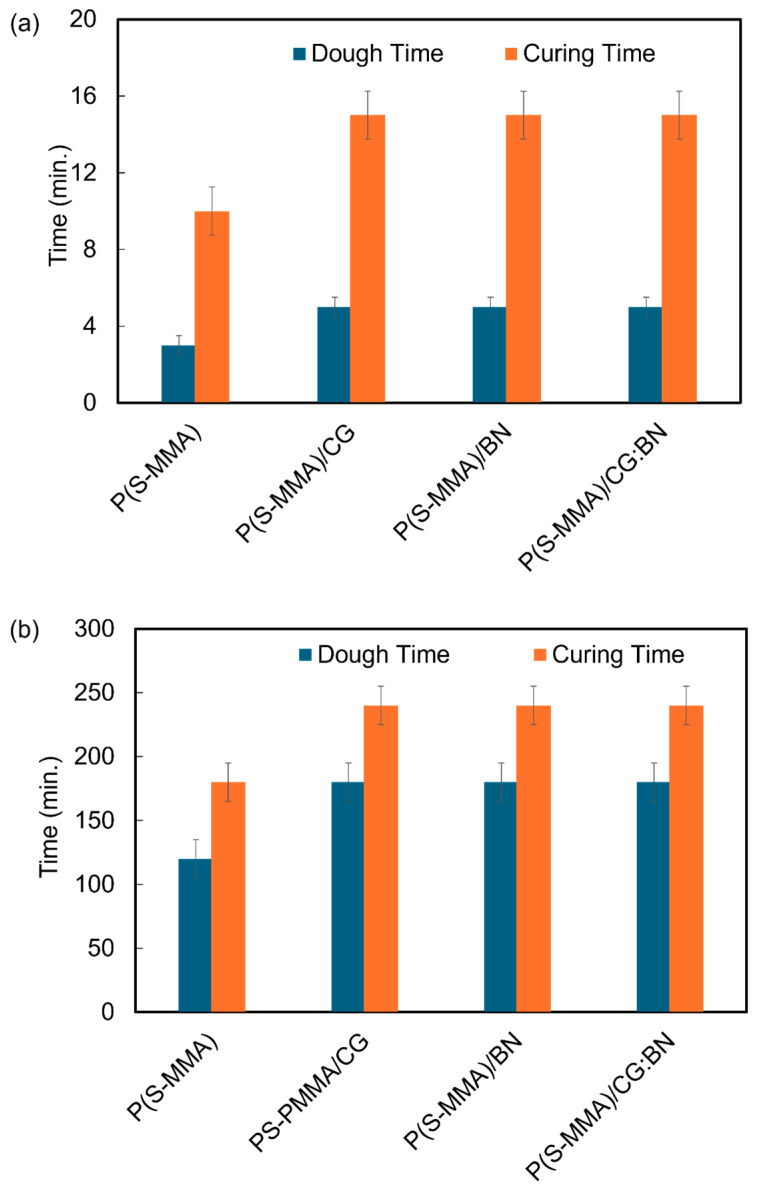
Curing and dough times of bone cement samples using different methods. (**a**) Suspension polymerization and (**b**) bulk polymerization.

Figure 9a,b show the dough and curing times of the bone cement samples prepared using suspension and bulk polymerization techniques, respectively. The experimental dough and curing times of the bone cement samples are summarized in Table 3. As evident from Figure 9a, the dough times of the P(S-MMA); P(S-MMA)/CG; P(S-MMA)/BN; and P(S-MMA)/CG:BN bone cement samples prepared using the suspension polymerization method are 2 min, 5 min, 5 min, and 5 min, respectively. Also, the curing times of the P(S-MMA); P(S-MMA)/CG; P(S-MMA)/BN; and P(S-MMA)/CG:BN bone cement samples prepared using the suspension polymerization method are 10 min, 15 min, 15 min, and 15 min, respectively. Likewise, the dough times of the P(S-MMA); P(S-MMA)/CG; P(S-MMA)/BN; and P(S-MMA)/CG:BN bone cement samples prepared using bulk polymerization method are 120 min, 180 min, 180 min, and 180 min, respectively. The curing times of the P(S-MMA); P(S-MMA)/CG; P(S-MMA)/BN; and P(S-MMA)/CG:BN bone cement samples prepared using the bulk polymerization method are 180 min, 240 min, 240 min, and 240 min, respectively. These results indicate that the bone cement samples based on polymer nanocomposites that were prepared using suspension polymerization have lower dough time (2–5 min) and faster curing kinetics (10–15 min) in comparison to the ones (180–240 min) with the bulk polymerization method. These faster curing kinetics in polymer nanocomposites prepared using suspension polymerization can be attributed to their shorter chain length and lower molecular weights. The shorter the polymer chain length, the faster the dough and curing time. At the same time, bulk polymerization results in relatively longer polymer chains and higher molecular weight, resulting in longer dough and curing times. These results of curing kinetics are in line with the thermal and nanomechanical characteristics of the samples. It is also important to note that the copolymer nanocomposites (P(S-MMA)–2D nanofiller nanocomposites) showed longer dough and curing times in comparison to that of their pure copolymer counterparts (P(S-MMA)), irrespective of their polymerization method. Incorporating 2D nanofillers into the copolymer matrix led to the impedance of the curing kinetics of the bone cement samples. Among the employed 2D nanofillers, the CG had less effect on the curing kinetics of the bone cement, while the other two nanofillers, BN and CG:BN, had higher and equal effects on the curing kinetics. This can be due to various physical interactions of the polymers with the 2D nanofillers, which causes a significant delay in the curing process compared to those without the nanofillers.

## 4. Conclusions

The methodological impact on the curing kinetics of P(S-MMA)–2D nanofiller nanocomposite bone cements that were prepared by bulk and suspension polymerization techniques have been investigated. P(S-MMA)–2D nanofiller nanocomposite bone cements were well-characterized for chemical, thermal, mechanical, and structural characteristics. The FE-SEM images revealed the non-porous structures of the cured P(S-MMA)–2D nanofiller nanocomposite bone, indicating that the implants would be long-lasting with higher mechanical strengths. The FT-IR results confirmed the successful formation of the P(S-MMA)–2D nanofiller nanocomposites prepared by both of the methods. The DSC results showed that the P(S-MMA)/CG:BN nanofiller nanocomposite has higher thermal stabilities than its copolymer counterparts. The nano-indentation results revealed that the elastic modulus of the P(S-MMA)/CG:BN (bulk polymerization) was as high as 7.89 GPa, and the hardness was found to be 0.219 GPa. Incorporating the 2D nanofiller (CG:BN) in the copolymer matrix synergistically enhances the thermo-mechanical properties of the bone cement samples. The P(S-MMA)/CG:BN that are prepared using suspension polymerization methods exhibit faster curing kinetics (15 min) than that of the ones prepared using the bulk polymerization (120–240 min) method. In conclusion, the nanocomposite samples prepared using bulk polymerization methods show higher mechanical characteristics, while the ones prepared using suspension polymerization methods show faster curing kinetics. However, it is still preferable that bone cement be prepared using the suspension polymerization method as it significantly reduces the risk of long-term exposure of patients to lethal monomers.

## Figures and Tables

**Table 1:** Thermal properties of P(S-MMA) and P(S-MMA)–2D nanofiller nanocomposites that are prepared using bulk and suspension polymerization methods.

Name of the Sample	Method	Glass Transition Temperature (T_g_; °C)
P(S-MMA)	Bulk	105.4
	Suspension	89.2
P(S-MMA)/CG	Bulk	105.1
	Suspension	98.6
P(S-MMA)/BN	Bulk	105.9
	Suspension	99.3
P(S-MMA)/CG:BN	Bulk	106.4
	Suspension	100.0

**Table 2:** Nanomechanical properties of P(S-MMA) and P(S-MMA)–2D nanofiller nanocomposites that are prepared using bulk and suspension polymerization methods.

Name of the Sample	Method	Elastic Modulus (GPa)	Hardness (GPa)
P(S-MMA)	Bulk	6.82	0.196
	Suspension	6.52	0.191
P(S-MMA)/CG	Bulk	6.46	0.189
	Suspension	6.36	0.181
P(S-MMA)/BN	Bulk	7.23	0.205
	Suspension	7.12	0.197
P(S-MMA)/CG:BN	Bulk	7.89	0.219
	Suspension	7.54	0.209

**Table 3:** Bone cement composition, their respective dough time, and curing time.

Name of the Sample	Method	Dough Time (min)	Curing Time (min)
P(S-MMA)	Bulk	120	180
	Suspension	2	10
P(S-MMA)/CG	Bulk	180	240
	Suspension	5	15
P(S-MMA)/BN	Bulk	180	240
	Suspension	5	15
P(S-MMA)/CG:BN	Bulk	180	240
	Suspension	5	15

## Data Availability

The data will be made available at the request of the corresponding author.
